# Squamous cell carcinoma of the bladder in a female associated with multiple bladder stones

**DOI:** 10.1186/1756-0500-6-354

**Published:** 2013-09-04

**Authors:** Jae Hyung Cho, Jean L Holley

**Affiliations:** 1 College of Medicine, University of Illinois at Urbana-Champaign, Urbana, IL, USA

**Keywords:** Squamous cell carcinoma, Urinary bladder calculi, Cystoscopy

## Abstract

**Background:**

Bladder cancer is the most common malignancy in the urinary tract. Urothelial carcinoma is the most common histologic type of bladder cancer in the United States, accounting for approximately 90%. Squamous cell carcinoma is less common, making up 3-5% of bladder cancers. We present a case of squamous cell carcinoma in a female associated with multiple bladder stones.

**Case presentation:**

A 76-year-old Caucasian woman presented to the emergency department with gross hematuria and dysuria for one month. Urinalysis showed many RBCs and WBCs with positive nitrite. She was admitted with an initial impression of urinary tract infection and intravenous ceftriaxone was started. Urine culture grew greater than 100,000 cfu/ml of Enterococcus species. Computed tomographic imaging of the abdomen/pelvis with oral contrast revealed a markedly distended bladder with hemorrhage, multiple calculi, and diffuse bladder wall thickening. Cystoscopy was performed for diffuse bladder wall thickening and demonstrated numerous bladder stones, a bladder mass, and organized blood clots. Biopsy of the mass was consistent with high-grade carcinoma with squamous differentiation. The bladder cancer was not surgically resectable and radical cystectomy was not recommended due to old age and poor functional status. The patient refused chemotherapy and she died in 6 months.

**Conclusions:**

The association between foreign bodies in the bladder and sqaumous cell carcinoma is well established. Long-standing bladder stones have been implicated as a cause of squamous cell carcinoma of the bladder. Our female patient’s unusual presentation with multiple bladder stones and sqaumous cell carcinoma of the bladder highlights the association between these two conditions.

## Background

Bladder cancer is the most common malignancy in the urinary tract. Urothelial carcinoma is the most common histologic type of bladder cancer in the United States, accounting for approximately 90%. Nonurothelial bladder cancers are less common, comprising approximately 5% of all bladder cancers. In parts of the world where infection with Schistosoma haematobium is prevalent, squamous cell carcinoma is the most common bladder cancer, responsible for approximately 50% of all bladder cancers [1]. In North America, however, squamous cell carcinoma is less common, making up only 3-5% of bladder cancers [2]. We present a case of squamous cell carcinoma of the bladder in a female associated with multiple bladder stones.

## Case presentation

**Figure 1:**
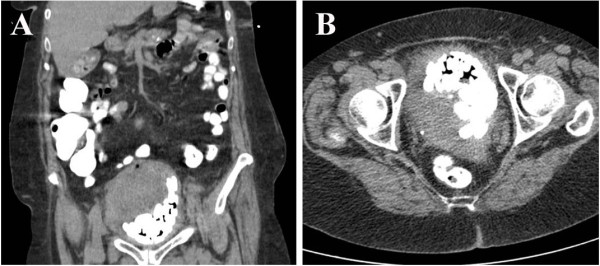
CT of the abdomen/pelvis with oral contrast, coronal view (A) and axial view (B).

**Figure 2:**
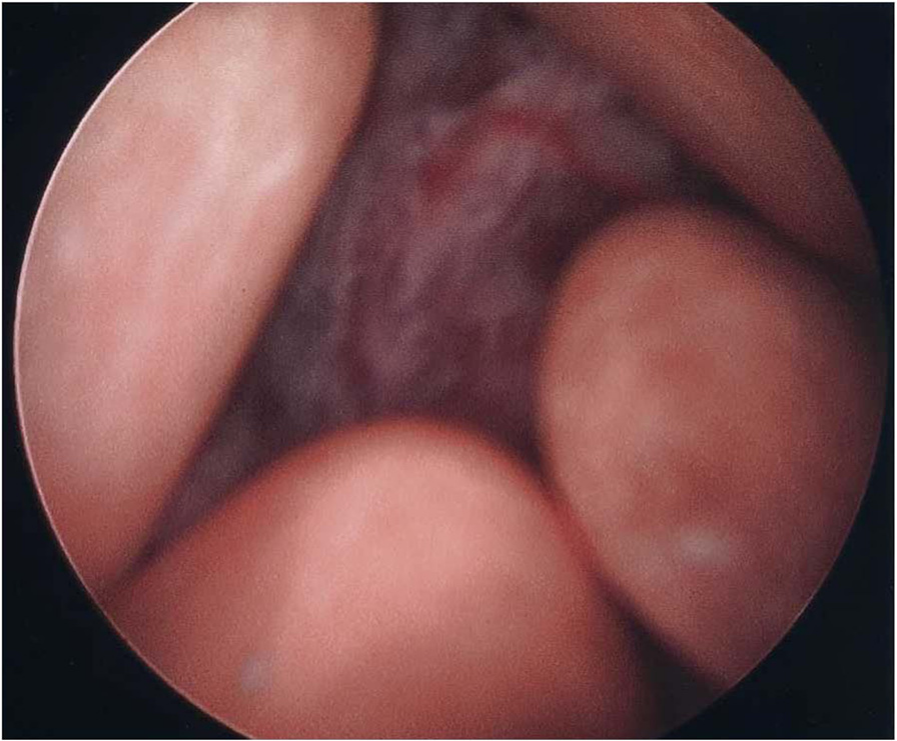
Cystoscopy showing a bladder mass and multiple calculi.

**Figure 3:**
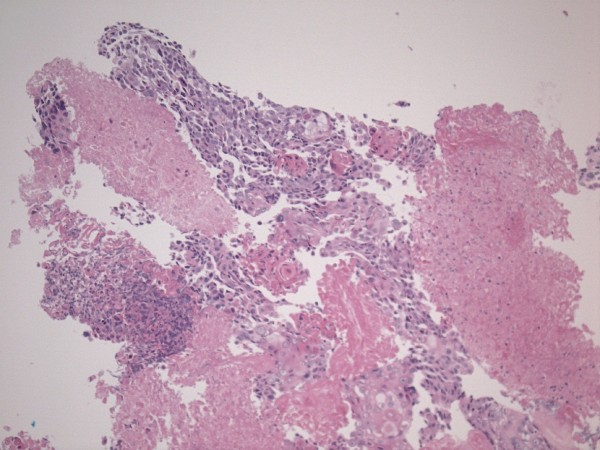
Biopsy of the mass (H&E, X100) showing high-grade carcinoma with squamous differentiation.

A 76-year-old Caucasian woman with no significant past medical history presented to the emergency department with gross hematuria and dysuria for one month. She also reported a sensation of bladder fullness, constipation, and 12–13 pounds of unintentional weight loss over the past 1.5 months. She was a former smoker who quit smoking many years ago. Vital signs were normal. Abdominal exam was significant for mild lower abdominal tenderness without rebound. Genital exam was unremarkable. Complete blood count was unremarkable. Comprehensive metabolic panel revealed sodium 122 mmol/L, blood urea nitrogen 16 mg/dL, creatinine 0.7 mg/dL, and glucose 130 mg/dL. Urinalysis showed many RBCs and WBCs with positive nitrite and 3+ protein with no crystals. She was admitted with an initial impression of urinary tract infection and intravenous ceftriaxone was started. Urine culture grew greater than 100,000 cfu/ml of Enterococcus species. Computed tomographic imaging of the abdomen/pelvis with oral contrast (Figure 1) revealed a markedly distended bladder with hemorrhage, multiple calculi, and diffuse bladder wall thickening. Cystoscopy (Figure 2) was performed for diffuse bladder wall thickening and demonstrated numerous bladder stones, a bladder mass, and organized blood clots. Clot removal and biopsy of the mass were performed. One small stone was irrigated out of the bladder and the biochemical analysis of the calculi revealed carbonate apatite. Biopsy of the mass was consistent with high-grade carcinoma with squamous differentiation (Figure 3). CT brain did not show any evidence of metastasis. The bladder cancer was not surgically resectable and radical cystectomy was not recommended due to old age and poor functional status. She was referred to medical oncologist but refused chemotherapy. She declined slowly thereafter and died in 6 months.

## Discussion

Bladder stones commonly occur in the setting of bladder outlet obstruction, genital prolapse, pelvic surgery, neurogenic bladder, or foreign bodies. In adults, bladder calculi rarely occur spontaneously in the absence of risk factors. The cause of our patient’s multiple bladder stones is unclear and unusual in a female patient. However, her squamous cell carcinoma can perhaps be attributed to multiple bladder stones. The association between chronic bladder irritation and squamous cell carcinoma has been postulated by many researchers [3,4]. Chronic bladder irritation includes chronic or recurrent urinary tract infection, chronic indwelling urinary catheter, bladder calculi, foreign bodies, intravesical Bacillus Calmette-Guerin (BCG) and prolonged exposure to cyclophosphamide [5]. Bladder cancer is associated with upper and lower urinary tract stones [4]. A 2-fold increase in bladder cancer risk was observed with a history of bladder stones in a case–control study [6]. Several studies also showed positive association between kidney or ureteral stones and the risk of bladder cancer [7,8]. Long-standing bladder stones have been implicated as a cause of squamous cell bladder cancer via chronic mucosal injury with resulting inflammation and disruption of the protective glycosaminoglycan layer [9]. Our patient’s unusual presentation with multiple bladder stones and squamous cell carcinoma highlights this association. The 5-year and 2-year overall survival rates have been reported as 10.6% and 47.6% respectively from M.D. Anderson Cancer Center [10]. Radical cystectomy remains the mainstay of therapy in select patients with resectable disease. Chemotherapy and radiation therapy can be considered in patients who are not surgical candidates or patients with metastasis.

## Conclusions

The association between foreign bodies in the bladder and sqaumous cell carcinoma is well established. Long-standing bladder stones have been implicated as a cause of squamous cell carcinoma of the bladder. Our female patient’s unusual presentation with multiple bladder stones and sqaumous cell carcinoma of the bladder highlights the association between these two conditions.

## Consent

Written informed consent was obtained from the patient for publication of this Case Report and any accompanying images. A copy of the written consent is available for review by the Editor-in-Chief of this journal.

## Competing interests

The authors declare that they have no competing interests.

## Authors’ contributions

JHC and JLH contributed to the writing of the manuscript. Both authors reviewed and approved the final version of the manuscript.
